# Supplementary material for: Rapid oxygen exchange between hematite and water vapor
Source: Nat Commun. 2021 Nov 10;12:6488. doi: 10.1038/s41467-021-26601-4 (PMC8580966; doi:10.1038/s41467-021-26601-4)
Supplement: Supplementary file 1 — Supplementary Information [file 41467_2021_26601_MOESM1_ESM.pdf]

Supplementary Information

Rapid oxygen exchange between hematite and water vapor

Authors

Zdenek Jakub[1]†, Matthias Meier[1,2], Florian Kraushofer[1], Jan Balajka[1], Jiri Pavelec[1], Michael Schmid[1], Cesare Franchini[2,3], Ulrike Diebold[1], Gareth S. Parkinson[1*]

Affiliations

[1]Insitute of Applied Physics, TU Wien, Vienna, Austria

[2]University of Vienna, Faculty of Physics and Center for Computational Materials Science, Vienna, Austria

[3] Alma Mater Studiorum - Università di Bologna, Bologna, Italy

†current affiliation: Central European Institute of Technology (CEITEC), Brno University of Technology, Czech Republic

*correspondence to: parkinson@iap.tuwien.ac.at

Supplementary Note 1

Corrections of the *m/e* = 18 signal in the TPD experiments

The TPD data shown in Fig. 2A,B of the main text are corrected for water ($H_2{}^{16}O$) adsorption from the background, the cracking pattern of the $H_2{}^{18}O$ and the $H_2{}^{16}O$ impurity in the $H_2{}^{18}O$ beam. The correction of the *m/e* = 18 signal is calculated by the following formula:

$$I_{18,\mathrm{corrected}} = I_{18,\mathrm{raw}} - f_{\mathrm{beam+crack}} * I_{20} - I_{18,\mathrm{blank}},$$

where $I_{18,\mathrm{raw}}$ is the raw data of the TPD scan (after subtraction of a constant offset due to higher *m/e* = 18 background), $f_{\mathrm{beam+crack}}$ is the correction factor for the $H_2{}^{16}O$ signal in the $H_2{}^{18}O$ beam, I_{20} is the *m/e* = 20 signal acquired simultaneously to $I_{18,\mathrm{raw}}$ and $I_{18,\mathrm{blank}}$ is the *m/e* = 18 signal measured in a blank experiment with no $H_2{}^{18}O$ dose (also with subtracted constant offset).

The plot of $I_{18,\mathrm{raw}}$, I_{20} and the smoothed $I_{18,\mathrm{blank}}$ signal is shown in Supplementary Figure 1A. The blank experiment was conducted in the same arrangement with the same sample treatment and preparation, just without the actual $H_2{}^{18}O$ dose. The nonzero *m/e* = 18 signal in a blank experiment is due to the background adsorption of $H_2{}^{16}O$ on the sample and the sample mount. The background is increased (in the 10^{-10} mbar range) prior to the TPD experiment due to the previous annealing of the sample in ${}^{16}O_2$ background, which is known to displace H_2O from the chamber walls.

The beam correction factor, $f_{\text{beam+crack}}$, accounts for the *m/e* = 18 signal coming from the $H_2{}^{18}O$ beam. It was determined from an experiment in which the $H_2{}^{18}O$ beam was directed into the chamber with the sample moved away, but the QMS ionizer moved near the beam trajectory. From this data, shown in Supplementary Figure 1B, it can be determined how much of the *m/e* = 18 signal is due to the cracking of $H_2{}^{18}O$ and $H_2{}^{16}O$ impurity in the beam. The $f_{\text{beam+crack}}$ was calculated as the ratio of the (*m/e* = 18) and (*m/e* = 20) signals averaged over the time when the beam shutter was opened (between 50 - 120 s in Fig. S1B). Depending on the constant background subtraction method, the value of $f_{\text{beam+crack}}$ lies between 0.11 (when signal minimum is taken as the background) and 0.05 (when the signal average before the beam shutter opening, 0 - 40 s, is taken as the background). For the data correction shown in the main text, a mean value of $f_{\text{beam+crack}} = 0.08$ was used.

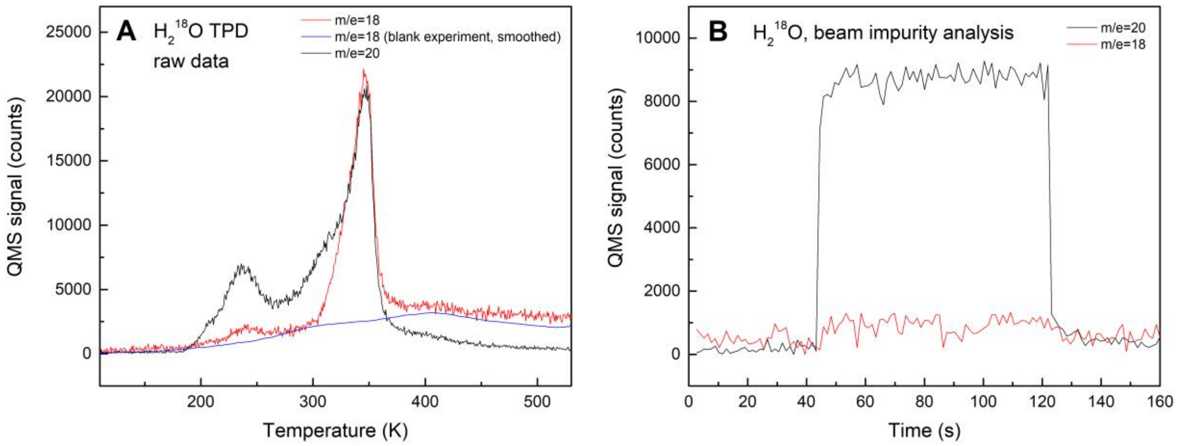

Supplementary Figure 1: Corrections of the *m/e* = 18 signal. A) Raw $H_2{}^{18}O$ TPD data showing simultaneously measured *m/e* = 20 and *m/e* = 18 signals. The blue curve shows an *m/e* = 18 signal measured in a blank experiment. In the processed data, this signal is subtracted from the *m/e* = 18 signal. B) Opening the $H_2{}^{18}O$ beam shutter into the mass spectrometer without the sample in sight shows the ratios of *m/e* = 20 and *m/e* = 18 signal. This is then used to correct the TPD signal for the cracking pattern and beam impurity. Source data are provided as a Source Data file.

Supplementary Note 2

Summary of all near-ambient-pressure and liquid-water experiments

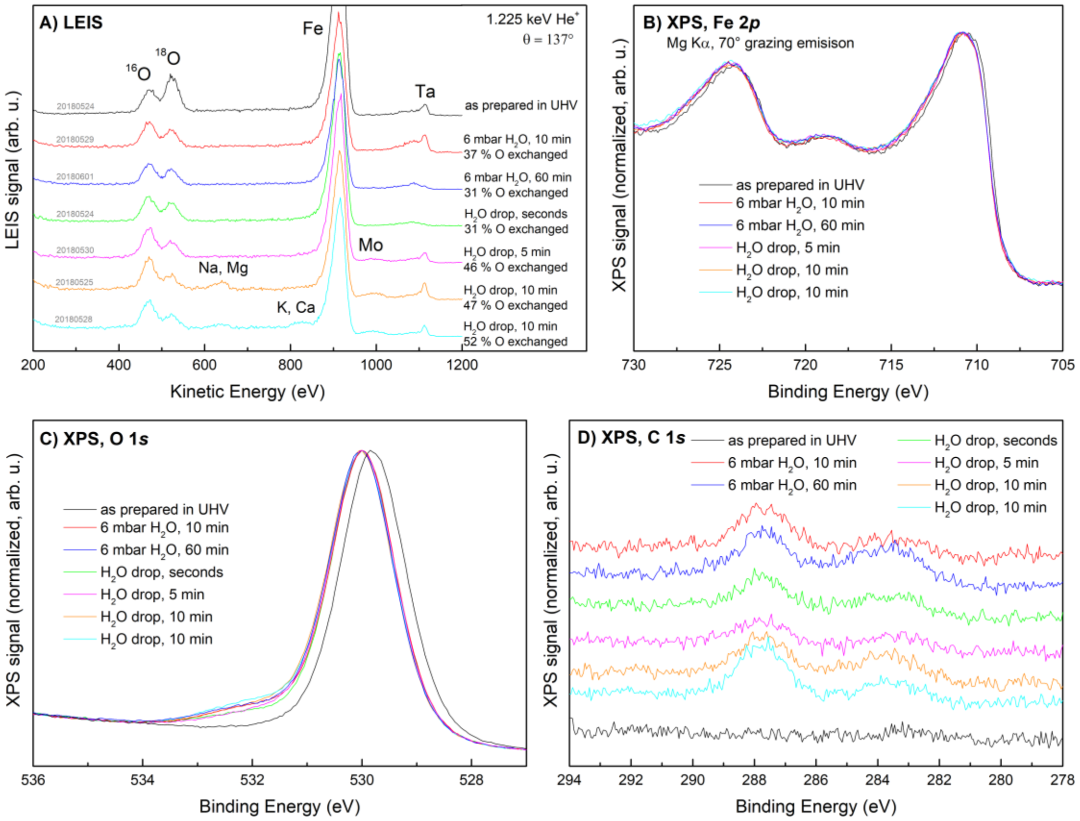

Supplementary Figure 2: LEIS and XPS characterization of all the near-ambient pressure and liquid-water exposure experiments carried out within this project. The LEIS spectra after prolonged liquid exposure (5 and 10 min) show higher fraction of ^{16}O, but also show additional peaks whose position is consistent with Na, Mg, K, Ca and Mo. XPS spectra of all these experiments show small carbonaceous signal and an O 1s shoulder attributed to carboxylic species. Importantly, the amount of carbonaceous contamination signal observed in the C 1s region does not correlate to the amount of exchanged O observed in LEIS. Source data are provided as a Source Data file.

Supplementary Note 3

Animations of the considered diffusion pathways

Supplementary Movies 1 and 2 show visualizations of the two considered surface diffusion pathways of water molecules on α-$Fe_2O_3(1\bar{1}02)$, labelled pathway A and pathway B in the main text. The color code is kept identical to the main text figure. For higher clarity, the surface atoms positions are kept constant. Adsorbates positions are according to the calculations, passing through NEB images, connected via a linear interpolation.

Models of structures along pathways A and B

All the structures shown in Figure 4 in the main text are included as .cif files in the Supplementary Data Files.

Supplementary Note 4

The effect of U_{eff} value and ZPE corrections on the computational results

The energies of transition states of pathways A and B computed using different values of U_{eff} and considering different corrections are listed in Supplementary Table 1. The main conclusions are not affected by the varying methodology.

Supplementary Table 1: Transition state energies (eV) calculated with different U_{eff} values and different corrections (vdW, ZPE).

	**U_{eff}=4** **D2-VdW**	**U_{eff} = 5** **D2-VdW**	**U_{eff} = 5** **no-VdW**	**U_{eff} = 5** **no-VdW**
				**with ZPE**
TS path A	0.780	0.796	0.880	0.891
TS path B	0.707	0.726	0.688	0.671
TS^A-TS^B	0.072	0.070	0.192	0.221
0 → B4/A6	-0.267	-0.288	-0.228	-0.168

Supplementary Note 5

Reaction rates of pathways A and B

Supplementary Figure 3 shows plots of reaction rates of pathways A and B using various computational methods. Comparison of panels A and B reveals that the exact choice of functional and treating of van der Waals interactions has a significant impact on the calculated rates, but it does not change the main conclusion that pathway B is preferred. Panel C shows rates calculated taking into account ZPE corrections of the relevant surface and adsorbate atoms involved in the diffusion process. By comparison to panel B, the ZPE corrections have surprisingly little effect on the calculated rates. The exchanged O_{surf}, lifted out of the surface plane shows similar changes in ZPE compared to O_{mol} atoms of molecules creating additional bonds when forming dimers. Panel D shows rates calculated using the vibrational entropies instead of the commonly assumed prefactor value of 10^{13} s^{-1}. Panel E is included for completeness, as due to transition states having only 3N−1 modes, calculating ΔS entities leads to a small error. To take this into account we added a 1D translational mode to the transition state. The characteristic length of choice was 5 Å and leads to a shift to higher temperature. The quality of such an approximation can be discussed, but doesn't affect our conclusions, as both mechanisms are of the same nature.

Overall, all the tested methods lead to the same conclusion that pathway B is preferred over pathway A.

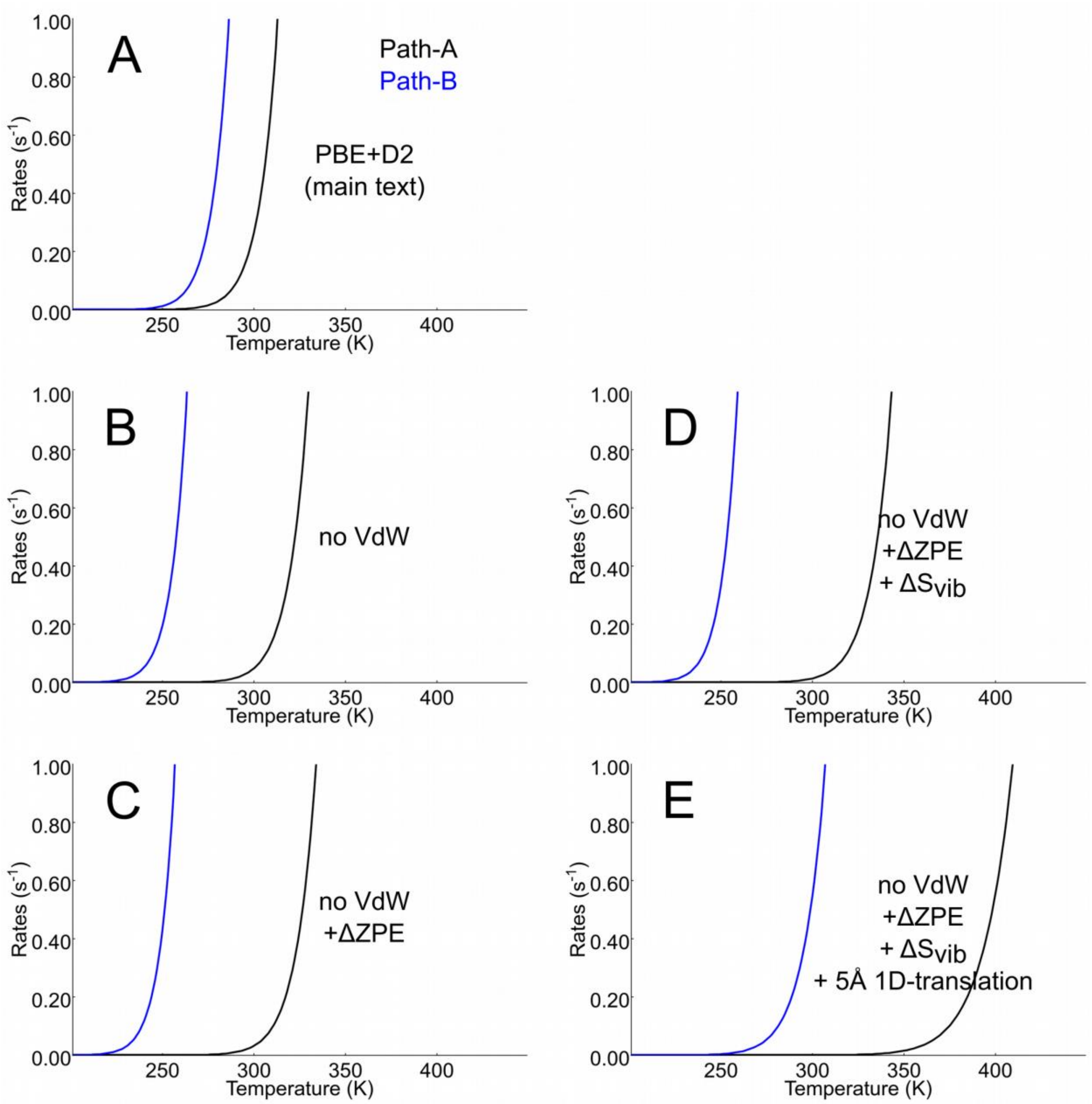

Supplementary Figure 3: Comparison of reaction rates for pathway A (black) and B (blue) using different assumptions/approximations. The rates are calculated: (A) assuming a prefactor of 10^{13} s^{-1} ($\Delta S = 0$) and using the PBE+D2 energies from the main text, (B) removing the VdW corrections, (C) adding ZPE corrections. (D) The respective vibrational entropies are estimated and used instead of the previous assumptions of ΔS=0, in addition to the ZPE corrections. (E) Due to the inconsistency regarding total modes between initial and transition state (3N vs 3N−1 modes), we added a 1D translational contribution to the transition state (entropy and enthalpy components), with a characteristic length of 5 Å. In all the tested cases, pathway B is preferred over pathway A.
